# Diurnal Profiles of Melatonin Synthesis-Related Indoles, Catecholamines and Their Metabolites in the Duck Pineal Organ

**DOI:** 10.3390/ijms150712604

**Published:** 2014-07-16

**Authors:** Bogdan Lewczuk, Natalia Ziółkowska, Magdalena Prusik, Barbara Przybylska-Gornowicz

**Affiliations:** Department of Histology and Embryology, Faculty of Veterinary Medicine, University of Warmia and Mazury in Olsztyn, Oczapowskiego Str. 13, 10-719 Olsztyn, Poland; E-Mails: ntrzaska@o2.pl (N.Z.); mprusik@gmail.com (M.P.); przybylb@gmail.com (B.P.-G.)

**Keywords:** pineal organ, diurnal rhythm, melatonin, serotonin, tryptophan derivatives, indoles, catecholamines, HPLC, domestic duck

## Abstract

This study characterizes the diurnal profiles of ten melatonin synthesis-related indoles, the quantitative relations between these compounds, and daily variations in the contents of catecholamines and their metabolites in the domestic duck pineal organ. Fourteen-week-old birds, which were reared under a 12L:12D cycle, were killed at two-hour intervals. The indole contents were measured using HPLC with fluorescence detection, whereas the levels of catecholamines and their metabolites were measured using HPLC with electrochemical detection. All indole contents, except for tryptophan, showed significant diurnal variations. The 5-hydroxytryptophan level was approximately two-fold higher during the scotophase than during the photophase. The serotonin content increased during the first half of the photophase, remained elevated for approximately 10 h and then rapidly decreased in the middle of the scotophase. *N*-acetylserotonin showed the most prominent changes, with a more than 15-fold increase at night. The melatonin cycle demonstrated only an approximately 5-fold difference between the peak and nadir. The 5-methoxytryptamine content was markedly elevated during the scotophase. The 5-hydroxyindole acetic acid, 5-hydroxytryptophol, 5-methoxyindole acetic acid and 5-methoxytryptophol profiles were analogous to the serotonin rhythm. The norepinephrine and dopamine contents showed no significant changes. The DOPA, DOPAC and homovanillic acid levels were higher during the scotophase than during the photophase. Vanillylmandelic acid showed the opposite rhythm, with an elevated level during the daytime.

## 1. Introduction

**Figure 1:**
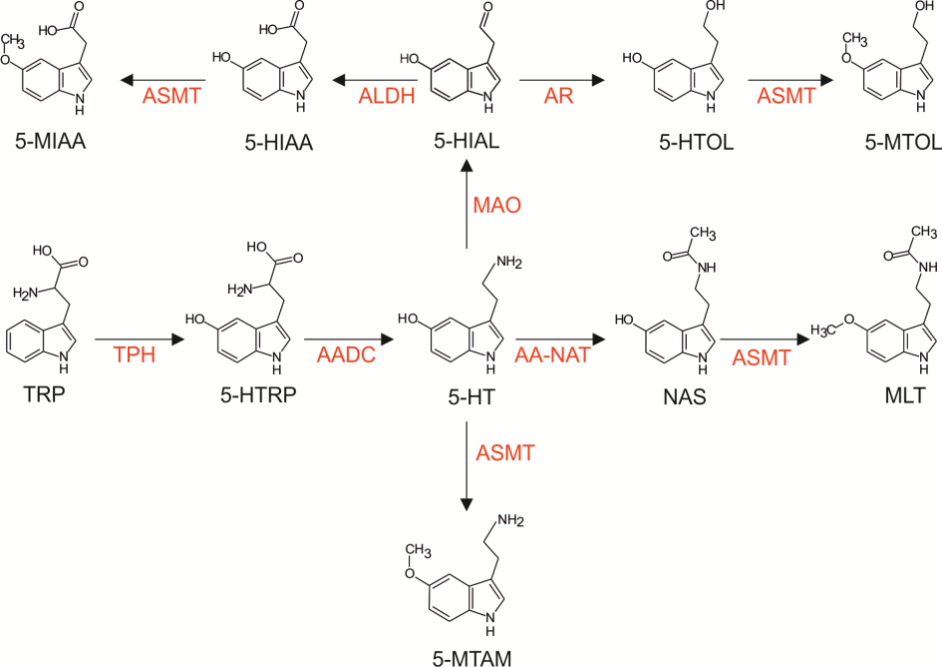
Synthesis of serotonin and its further metabolism in the pineal organ. Abbreviations: TPH, tryptophan hydroxylase; AADC, aromatic l-amino acid decarboxylase; AA-NAT, arylalkylamine *N*-acetyltransferase; ASMT, *N*-acetylserotonin *O*-methyltrans-ferase; MAO, monoamine oxidase; AR, aldose reductase; ALDH, aldehyde dehydrogenase; TRP, tryptophan; 5-HTRP, 5-hydroxytryptophan; 5-HT, serotonin; NAS, *N*-acetyl-serotonin; MLT, melatonin; 5-HIAL,5-hydroxyindole acetic aldehyde; 5-HIAA, 5-hydroxyindole acetic acid; 5-HTOL, 5-hydroxytryptophol; 5-MIAA, 5-methoxyindole acetic acid; 5-MTOL, 5-methoxytryptophol; 5-MTAM, 5-methoxytryptamine.

Indole metabolism in the pineal organ (Figure 1) commences with the active uptake of the essential amino acid tryptophan (TRP) into parenchymal cells [1,2,3]. Next, TRP is hydroxylated by tryptophan hydroxylase to 5-hydroxytryptophan (5-HTRP), which is decarboxylated by aromatic amino acid decarboxylase to serotonin (5-HT). This indoleamine is a starting point for several metabolic pathways. Among these pathways, the most investigated one is the transformation of 5-HT into melatonin (MLT). This process includes two steps: acetylation by arylalkylamine *N*-acetyltransferase (AA-NAT) to *N*-acetylserotonin (NAS) and methylation by *N*-acetylserotonin *O*-methyltransferase (ASMT) to MLT. The pineal hormone is released from cells immediately after synthesis. Other indolic compounds, such as 5-hydroxyindole acetic acid (5-HIAA) and 5-hydroxytryptophol (5-HTOL), are produced after the oxidative deamination of 5-HT. The methylation of 5-HIAA and 5-HTOL leads to the formation of 5-methoxyindole acetic acid (5-MIAA) and 5-methoxytryptophol (5-MTOL), respectively. Moreover, 5-HT can be directly methylated by ASMT to 5-methoxytryptamine (5-MTAM).

In the pineal organs of almost all species, MLT is produced according to a diurnal rhythm, with prominently elevated synthesis at night [1,2,4,5]. The processes controlling this rhythm differ significantly among vertebrate classes and even among the species within a class [6,7,8,9,10]. Despite divergences, the most common and conserved features of the melatonin-rhythm generating system include a control by environmental light and a dependence on the circadian clock [6,7].

The mechanisms regulating MLT secretion in the avian pineal organ are highly complex, most likely because of the intermediate phylogenetic position of this organ. Similar to lower vertebrates, pinealocytes in birds contain photosensitive molecules and can directly receive environmental light [11,12]. Moreover, these cells possess an endogenous oscillator that generates a rhythm of MLT synthesis [11,13]. In addition, the pineal activity in birds, similar to mammals, is also controlled by light acting on the retina and by the circadian clock in the suprachiasmatic nucleus [14,15]. The integrated signals from the retina and from the hypothalamus are transmitted to the pineal organ via the sympathetic innervation [16]. In contrast to the mammalian pineal gland, norepinephrine (NE), which is released from the autonomic nerve fibers, inhibits melatonin secretion in avian pinealocytes [17]. The mechanisms regulating the pineal activity in birds have been investigated in only a few species. The obtained data show important interspecies differences [18,19], including the variable significance of the sympathetic input [20]. 

Thus far, studies concerning the diurnal rhythm of pineal indole metabolism focused almost exclusively on MLT and on two enzymes responsible for its synthesis: AA-NAT and ASMT. The latter enzyme was less intensively studied than AA-NAT because ASMT has been long regarded as having little importance in the regulation of MLT synthesis. In contrast to MLT, the contents of other indolic compounds were seldom investigated, primarily due to the limitations related to analytical methods. Currently, the most effective techniques for measuring 5-hydroxyindoles and 5-methoxyindoles are HPLC methods [21] with electrochemical [22,23,24,25,26] or fluorescence detection [27,28,29,30,31]. Fluorescence detection has been less frequently used in pineal studies than electrochemical detection [21], usually for assays of melatonin only [29] or after derivatization procedures [30,31]. The considerable problems with using HPLC methods in pineal research include the following: (1) large differences in the pineal contents of various indoles, complicating fluorometric detection; (2) insufficient sensitivity to measure all essential compounds in the pineal organ over a 24-h-cycle; and (3) large differences in the polarity and retention times between 5-hydroxyindoles and 5-methoxyindoles, causing difficulties in the simultaneous detection of both groups of indoles within a single run.

Our knowledge concerning the indole contents in the avian pineal organ is almost exclusively limited to MLT and 5-MTOL, which have been measured in several species by radioimmunoassays [32,33,34]. The data concerning other pineal indoles are only fragmentary [35,36,37].

The first aim of this study was to characterize the diurnal rhythms of MLT synthesis-related indoles contents and the quantitative relationships between these compounds in the pineal gland of the domestic duck. For this purpose, we developed a novel HPLC method based on gradient separation and fluorescence detection, with time-programmable detector sensitivity switching. This method enables the simultaneous analysis of ten indoles with extremely low quantification limit values. The second aim was to analyze changes in the pineal sympathetic input activity occurring over a period of 24 h. This goal was achieved by measuring catecholamines (norepinephrine, NE; dopamine, DA; 3,4-dihydroxy-l-phenylalanine, DOPA) and their metabolites (3,4-dihydroxyphenylacetic acid, DOPAC; homovanillic acid, HVA; vanillylmandelic acid, VMA) using HPLC with electrochemical detection.

## 2. Results and Discussion

### 2.1. Characteristics of Chromatographic Assays

**Figure 2:**
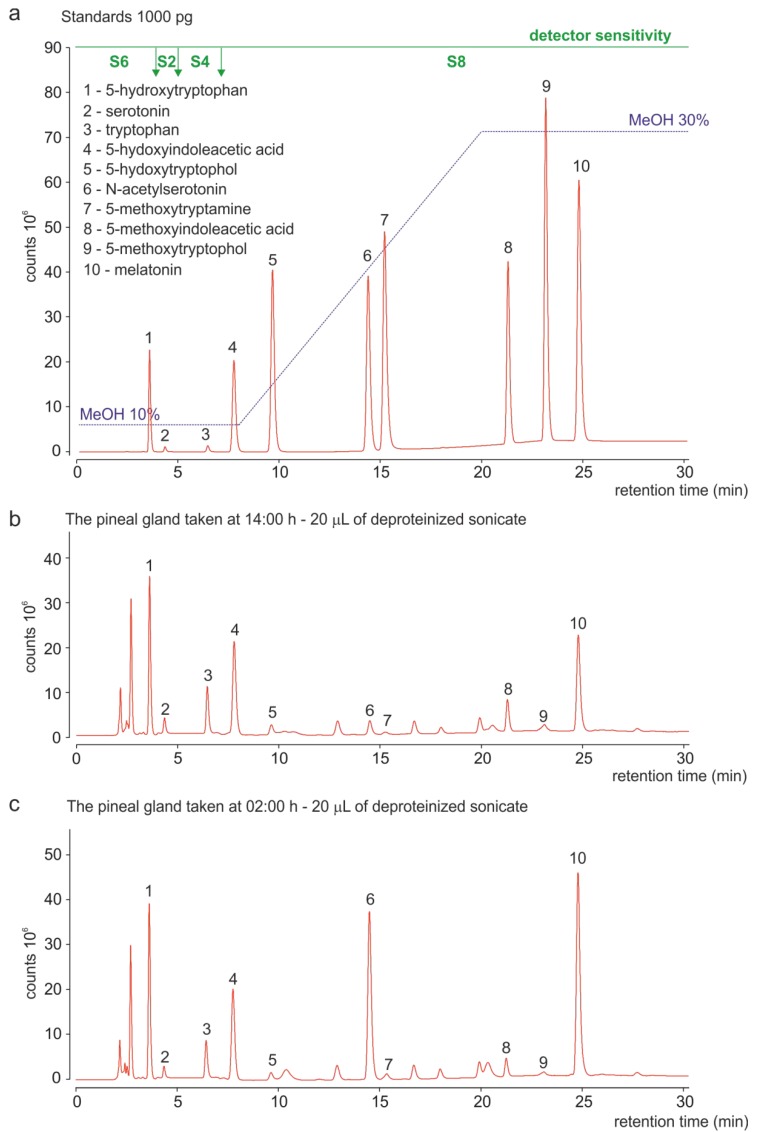
Chromatograms obtained using HPLC with fluorescence detection. (**a**) Separation of synthetic indoles applied as standards (1000 pg per injection); (**b**) Analysis of the duck pineal homogenate prepared from the organ taken at 14:00 h (20 µL per injection); (**c**) Analysis of the duck pineal homogenate prepared from the organ taken at 02:00 h (20 µL per injection). Green bar, switches of detector sensitivity.

In the present study, we proposed and validated a novel HPLC method with fluorescence detection for the simultaneous determination of ten MLT synthesis-related indoles in the pineal organ. The chromatograms of standards and pineal homogenates obtained using this method are shown in Figure 2. All peaks were well-separated, well-defined, and free from tailing and splitting. The calibration curves were linear (*r*^2^ = 0.999), ranging from 1 to 1000 pg per injection for all assayed indoles, excluding 5-HT and TRP. Due to their high content in the pineal organ, 5-HT and TRP were assayed using a lower sensitivity detector setting, and the calibration curves for these indoles were linear (*r*^2^ = 0.999) in the ranges of 5–10,000 pg per injection and 10–10,000 pg per injection, respectively. The limits of detection (S/N ratio of 3:1) for 5-HTRP, 5-HTOL, NAS, 5-MTAM, 5-MIAA, 5-MTOL and MLT were lower than 0.5 pg per injection, and the limits of quantification (S/N ratio of 10:1 and RSD ≤ 15%) for these compounds were established at 2 pg per injection. The limits of quantification for 5-HT, TRP and 5-HIAA were 10, 20 and 5 pg per injection, respectively.

The assay validation included the analysis of all critical points of the method at both the sample preparation and chromatographic steps. The first two questions concerned (1) the effect of perchloric acid, which was used as a protein precipitation agent during the sample preparation; on the indole content and (2) the stability of indoles in the tissue homogenate. The performed experiments showed that the levels of indoles, except for 5-MIAA and MLT, were similar (RSD < 5%) in the pineal homogenates deproteinized by perchloric acid precipitation and by ultrafiltration through a molecular filter with a 3-kDa molecular weight cut-off. The 5-MIAA and MLT contents were lower after ultrafiltration compared with acid precipitation by 35% ± 5% and 62% ± 5%, respectively. These differences were most likely caused by indoles binding to the filter membrane or to sample proteins. The concentrations of all assayed indoles did not change significantly after the six-hour storage of pineal homogenates at room temperature. The longer delay between the sample preparation and the chromatographic assay resulted in a decrease in the measured levels of indoles by 5%–47%, depending on the compound studied. Considering these data, the workflow was organized in a manner ensuring that the time between the sample preparation and the chromatographic assay did not reach four hours.

Routine validation tests were performed at the chromatographic step. To ensure the proper identification of compounds and to exclude peak overlapping, the gradient slope was modified over a wide range, and these changes invariably had identical effects on the analyzed peaks in the chromatograms of standard solutions and pineal homogenates. The assay showed extremely high intra-day and inter-day repeatability (Table 1). The exogenous indoles added to the samples were quantitatively recovered (Table 2). The 5-hydroxyindole levels measured using HPLC methods with fluorescence detection and with electrochemical detection did not differ by more than 12.5%.

**Table 1:** Intra-day and inter-day precision of the indole assay using HPLC with fluorescence detection. The standard solution containing 25 ng of each compound in 1 mL of 0.1 M perchloric acid was divided into aliquots and stored at −80 °C. After thawing, 10 µL of the solution was injected with ten replicates on the same day or once a day during ten consecutive days. The retention times and the peak areas were determined using Chromeleon 6.8 software (Dionex, Sunnyvale, CA, USA).

Peak No.	Compound	Intra-Day Precision		Inter-Day Precision	
		Retention Time MIN–MAX	Peak Area RSD (%)	Retention Time MIN–MAX	Peak Area RSD (%)
1	5-HTRP	3.453–3.455	1.8	3.451–3.470	2.2
2	5-HT	4.240–4.244	1.9	4.240–4.248	2.5
3	TRP	6.295–6.297	2.2	2.293–6.306	2.8
4	5-HIAA	7.654–7.656	2.4	7.650–7.770	3.2
5	5-HTOL	9.120–9.145	2.7	9.100–9.157	3.7
6	NAS	14.200–14.203	2.4	14.192–14.205	2.6
7	5-MTAM	15.200–15.208	2.1	15.197–15.230	3.2
8	5-MIAA	21.270–21.314	2.4	21.240–21.327	3.5
9	5-MTOL	23.057–23.067	2.8	23.054–23.087	3.4
10	MLT	24.604–24.607	2.5	24.600–24.630	3.7

**Table 2:** Recovery of indoles added to the pineal homogenate in the assay using HPLC with fluorescence detection. Five pineal organs were sonicated in 500 µL of perchloric acid and centrifuged at 1000× *g* for 5 min, and the supernatant was divided into portions. The solutions containing indoles at concentrations corresponding to 0, 25, 50, 250 and 500 pg on the column were added to these portions. Next, the samples were assayed as described in the “Experimental Section”. The test was repeated three times.

Peak No.	Compound	Parameters	Indole Added (pg on Column)			
			25	50	250	500
1	5-HTRP	Mean recovery (%)	102.3	99.2	100.2	101.2
		RSD (%)	3.2	2.2	1.8	2.0
2	5-HT	Mean recovery (%)	99.8	101.2	102.3	99.2
		RSD (%)	2.8	3.2	2.8	2.9
3	TRP	Mean recovery (%)	102.2	99.8	100.2	99.8
		RSD (%)	3.4	2.5	2.2	3.2
4	5-HIAA	Mean recovery (%)	98.8	100.6	100.5	99.8
		RSD (%)	2.9	3.2	1.9	2.8
5	5-HTOL	Mean recovery (%)	102.4	100.1	100.2	98.7
		RSD (%)	1.7	2.2	1.9	2.4
6	NAS	Mean recovery (%)	99.8	101.7	98.8	102.3
		RSD (%)	2.1	1.2	1.2	2.4
7	5-MTAM	Mean recovery (%)	99.7	100.1	101.4	102.4
		RSD (%)	1.7	2.4	2.7	2.8
8	5-MIAA	Mean recovery (%)	102.1	101.2	102.3	99.8
		RSD (%)	2.7	2.7	2.6	2.4
9	5-MTOL	Mean recovery (%)	101.7	98.5	99.5	97.5
		RSD (%)	1.9	2.3	2.4	3.2
10	MLT	Mean recovery (%)	103.2	100.1	1002	99.5
		RSD (%)	2.6	2.1	1.9	2.4

The advantages of our method include minimal sample preparation, high sensitivity, extremely good accuracy and repeatability. The use of gradient separation resolved the problem of large differences in the polarity and retention times between 5-hydroxyindoles and 5-methoxyindoles. A highly sensitive detector with time-programmable sensitivity switching enabled the simultaneous measurement of indoles occurring at extremely high (TRP, 5-HT) and extremely low (5-MTAM, 5-MTOL) levels. The proposed analytical method could also be easily adapted to other biological matrices. The minor disadvantage of our assay is the duration of a single analysis; however, the throughput of the method could be markedly increased by transferring the assay to UPLC conditions, which, in our opinion, should be easy to perform.

**Figure 3:**
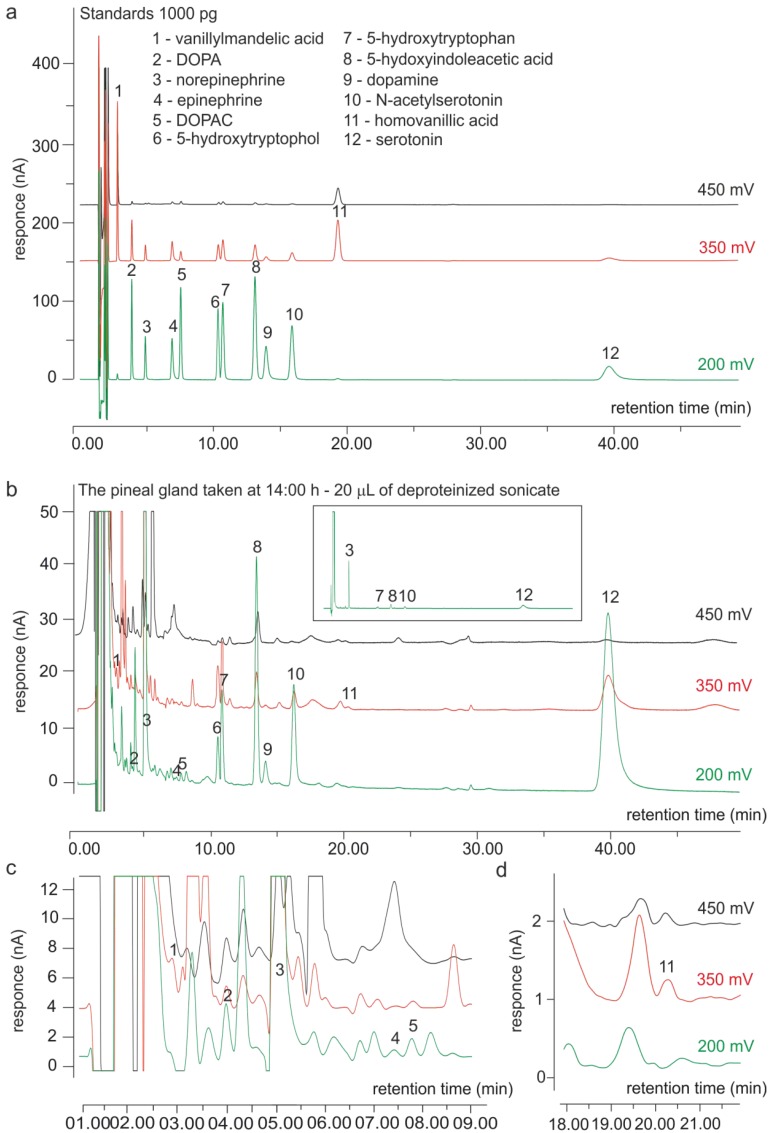
Chromatograms obtained using HPLC with electrochemical detection. (**a**)Separation of synthetic compounds applied as standards (1000 pg per injection); (**b**–**d**) Analysis of the duck pineal homogenate prepared from the organ taken at 14:00 h (20 µL per injection). For visualizing all peaks, different time and response scales were used in panels **b**, **c**, and **d**.

The assay of catecholamines in the duck pineal organ was performed using a modified, well-known HPLC method with electrochemical detection, employing a highly efficient, multi-electrode coulometric cell [24,38]. The chromatograms of standards and of the pineal homogenate obtained using this method are shown in Figure 3. The catecholamines and their metabolites were well separated from 5-hydroxyindoles. The calibration curves for DOPA, DA, NE, DOPAC, HVA and VMA were linear (*r*^2^ = 0.999), ranging from 5 to 50,000 pg per injection, and the responses for the lowest amounts of standards exceeded the criteria established for the determination of the limit of quantification (10-fold S/N ratio and CV ≤ 15%).

### 2.2. Pineal Indoles—Diurnal Rhythms and Quantitative Relations

#### 2.2.1. Serotonin and Its Precursors

**Figure 4:**
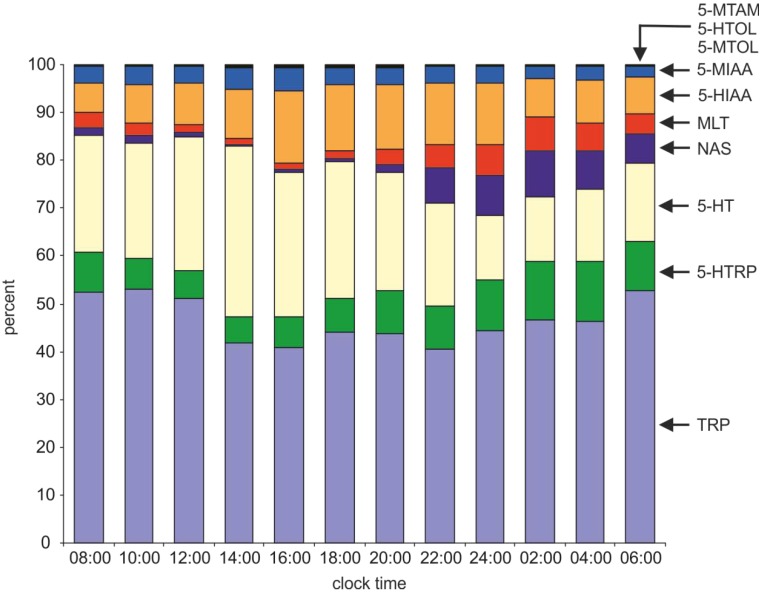
Percentage of indolic compounds measured in duck pineal organs taken at two-hour intervals over a daily cycle. The diagram demonstrates diurnal changes in the stoichiometric relationships between indoles (the input data are expressed as pmol/100 µg of protein).

**Figure 5:**
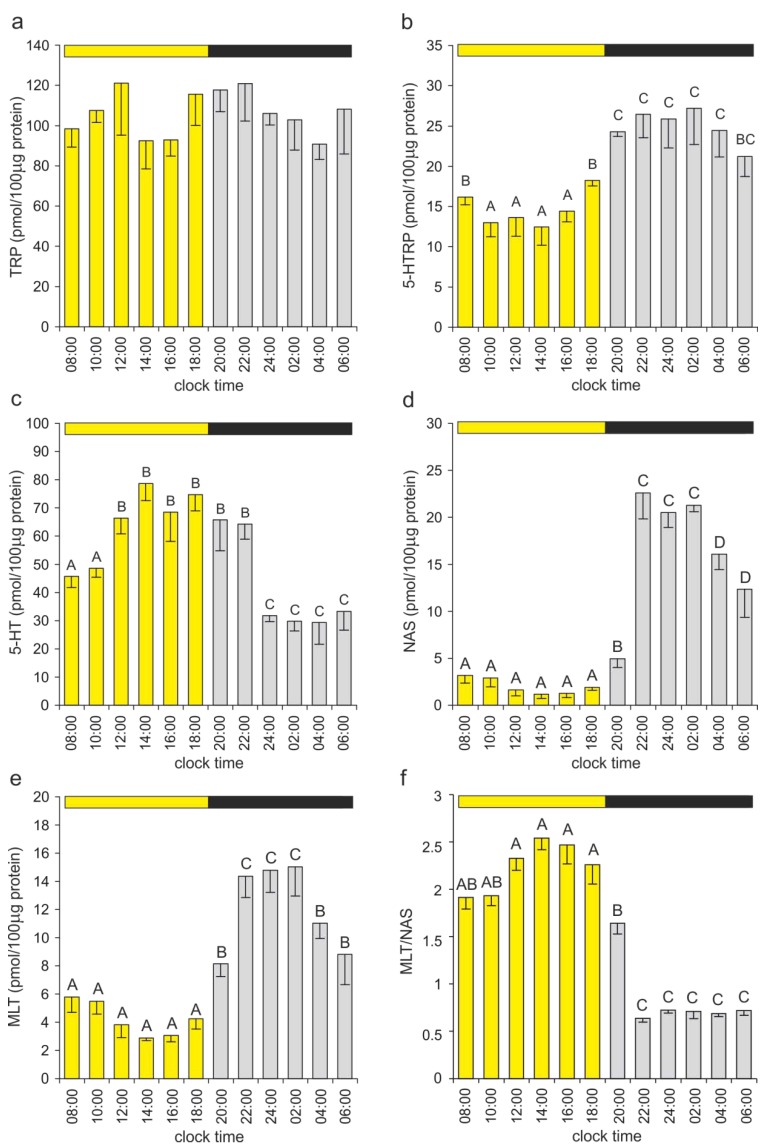
Indoles of the melatonin synthesis pathway. (**a−e**) The contents of TRP, 5-HTRP, 5-HT, NAS and MLT and (**f**) the MLT/NAS ratio in duck pineal organs taken at two-hour intervals over a daily cycle (means and standard errors, *n* = 5). The values signed with different letters are significantly different at *p* ≤ 0.05. Bars show the duration of the photophase (in yellow) and of the scotophase (in black).

TRP represented 40.6%–53.2% of the investigated indoles in the duck pineal gland (Figure 4), and its content did not change significantly during the course of the light-dark cycle (Figure 5a). High levels of TRP, with no differences between the mid-day and mid-night values, were also reported in the chicken pineal gland [39]. Because the TRP content greatly exceeded the level of its immediate derivate, 5-HTRP (Figure 4), it seems that the availability of this amino acid does not limit pineal indole metabolism under physiological conditions. However, this conclusion should be carefully interpreted because TRP is also used by pineal cells for protein biosynthesis. The experimental administration of TRP had variable effects on pineal indole synthesis, depending on the species studied and experimental conditions. In the Indian spotted owlet, the oral administration of TRP increased the pineal weight and the plasma MLT level during the breeding season, when the pineal is inactive, but not during the reproductively quiescent phase, when the pineal is active [40]. The administration of TRP in sheep did not change the pineal content of 5-HT, despite producing a five-fold elevation of the TRP content [41]. In contrast, rats treated with TRP displayed increased pineal levels of 5-HTRP and 5-HT [42,43].

In contrast to TPR, the 5-HTRP content in the duck pineal organ showed a diurnal rhythm, with the nighttime values approximately two-fold higher than the daytime values (Figure 5b). The level of this compound began to increase before the onset of darkness (at 18:00 h), suggesting the involvement of endogenous oscillator(s) in the regulation of its content. To our knowledge, the present study is the first to show the complete daily profile of 5-HTRP in the avian pineal organ. Piesiewicz *et al*. [39] measured the 5-HTRP level in the chicken pineal organ at two points of the daily cycle and demonstrated approximately two-fold higher content of this compound in the middle of the scotophase than in the middle of the photophase. Data concerning the diurnal changes in the 5-HTRP level in the pineal organs of other vertebrate classes are also scarce [25,44,45]. The daily rhythm of 5-HTRP, with a 30% increase in content during the scotophase with respect to the level at midday, was reported in the pineal organ of rainbow trout housed under a 12L:12D cycle but not under 16L:8D and 8L:16D cycles [25]. The 5-HTRP level did not show rhythmical variations in the pineal gland of Syrian hamsters kept under 14L:10D and 10L:14D cycles [44]. A nighttime increase in the 5-HTRP content was noted in Djungarian hamsters housed under a long photoperiod, 16L:8D, but not under a short photoperiod, 14L:10D [45]. No significant daily changes in the 5-HTRP level were found in Djungarian hamsters kept under natural photoperiods [46].

Our data concerning the diurnal rhythm of the 5-HTRP level in the duck pineal gland are consistent with studies regarding tryptophan hydroxylase in the chicken pineal organ, which indicated higher expression and activity of this enzyme at night than during the day [39]. A circadian oscillator and cAMP are considered the primary factors responsible for the control of tryptophan hydroxylase activity in the avian pineal organ [47,48,49].

From a quantitative point of view, 5-HT was the second compound among the investigated indoles in the duck pineal gland (Figure 4). Its level showed a prominent diurnal rhythm; the 5-HT content increased during the first half of the photophase, then remained at a high level for approximately 10 h, and finally rapidly decreased immediately before the middle of the scotophase (Figure 5c). The high content and diurnal rhythm of 5-HT are frequently mentioned features of the pineal organ in all vertebrate classes; however, the daily fluctuations in the level of this indoleamine have only been thoroughly characterized in a limited number of species, and the obtained results reveal conspicuous differences [23,24,25,26,44,45,46]. Among birds, the diurnal rhythm of the pineal 5-HT content has been described in the chicken [36], pigeon [35] and Japanese quail [50]. In the chicken pineal gland, the daily course of the 5-HT level had a bimodal pattern, with peaks at dawn and at dusk [36]. In the pigeon and Japanese quail, the 5-HT content reached its peak shortly after the onset of the photophase and reached the nadir in the middle of the scotophase [35,50]. Day-night fluctuations in the 5-HT level were noted in 2-week-old chickens hatched in summer but not in winter [39]. Notably, during both seasons chickens were kept under strictly controlled laboratory conditions, with 12-h-long photoperiod, therefore the authors attributed the difference in presence of diurnal changes in 5-HT level to maternal influence on the early postembryonic pineal gland development [39]. This phenomenon was also observed in the studies dealing with other parameters of the MLT-synthesis pathway in the chicken pineal organ [51,52]. Regarding other vertebrate classes, in the rainbow trout, a pineal 5-HT peak was noted immediately after the light to dark transition, followed by a severe reduction of this biogenic amine level at midnight [25]. The rhythm of the pineal 5-HT level occurred in fish kept under short (8L:16D) and neutral (12L:12D) photoperiods but not under long (16L:8D) photoperiods [25]. Similarly, photoperiod-dependent differences in the course of the pineal 5-HT rhythm were reported in the Djungarian hamsters [45,46]. In the Syrian hamster, the highest levels of pineal 5-HT were noted during the second portion of the photophase and at the beginning of the scotophase [23].

Special attention should be given to the stoichiometric relationship between TRP, 5-HTRP and 5-HT (Figure 4). The 5-HTRP level in the duck pineal organ was several times lower than the TRP content. The 5-HT/5-HTRP ratio ranged from 2.4 to 6.3 between 08:00 and 22:00 h but decreased to 1.1–1.2 at 24:00, 02:00 and 04:00 h. This decline was clearly caused by the following two factors: (1) the decrease in the 5-HT content due to its intensive transformation into NAS (see below) and (2) the nighttime increase in 5-HTRP synthesis. Our data regarding the levels of TRP, 5-HTRP and 5-HT led to the following conclusions: (1) hydroxylation of tryptophan should be considered an important step regulating pineal indole metabolism; (2) the nighttime rise in the 5-HTRP content is crucial to ensure sufficient 5-HT for intensive nocturnal NAS synthesis; and (3) 5-HTRP does not form a reserve pool for the synthesis of other indoles.

#### 2.2.2. Direct Derivatives of Serotonin

The metabolism of 5-HT in the pineal organ includes the following three primary pathways: (1) acetylation by AA-NAT to NAS; (2) oxidative deamination catalyzed by monoamine oxidase, leading to the formation of 5-hydroxyindole acetaldehyde, which is converted to 5-HIAA and to 5-HTOL by aldehyde dehydrogenase and by aldose reductase, respectively; and (3) methylation to 5-MTAM by ASMT (Figure 1).

The NAS content showed the most prominent diurnal rhythm among the investigated indoles in the duck pineal organ (Figure 5d). The nighttime levels of NAS (between 22:00 and 02:00 h) were approximately 16-fold higher than the daytime values (at 14:00 and 16:00 h). Data concerning the content of this indoleamine in other avian species are scare [35,36]. In the chicken pineal organ, NAS was undetectable during the light phase, and its level increased to approximately 1 ng/organ during the dark phase of the daily cycle [36]. A three-fold elevation in the NAS content at night was reported in adult pigeons [35].

NAS formation is generally considered the primary factor responsible for the diurnal changes in the 5-HT level in the pineal organ. This idea is supported by the results of experiments showing a negative correlation between 5-HT and NAS contents [44,45,46,53]. The decrease in the 5-HT level during the scotophase is usually interpreted as an effect of increased utilization of this indoleamine by AA-NAT, whose activity is markedly elevated at night [53,54]. This negative relationship between 5-HT and NAS was also observed in our study (Figure 4, and Figure 5c,d). Notably, the NAS level reached the maximum value at 22:00 h, whereas a drop in the 5-HT content was noted at 24:00 h. This delay suggests that the 5-HT content in the duck pineal organ was large enough to support the increased utilization of this indoleamine by the acetylation process at the beginning of night. When the 5-HT reserve pool had been consumed, then the 5-HT level declined. The rates of 5-HT synthesis and metabolism were most likely similar during the second portion of the night. At this time, 5-HT synthesis seems to be critical for ensuring the availability of this substrate for NAS and MLT production. As noted above, we showed a significant increase in the pineal 5-HTRP level during the scotophase, which may indicate that 5-HT synthesis is up-regulated at night. The increased synthesis of 5-HT during the scotophase has also been suggested by studies performed in rainbow trout [25] and in rodents [54,55], showing an elevated 5-HT level at the beginning of night, immediately before the onset of MLT synthesis. Notably, the results of some investigations have provided arguments against the crucial role of acetylation in generating the pineal 5-HT rhythm. In the Syrian hamster, a drop in the 5-HT level occurred before the increase in AA-NAT activity and in MLT content [23]. In the Chinese hamster, the highest levels of 5-HT, NAS and MLT were noted during the photophase and coincided with each other [56].

**Figure 6:**
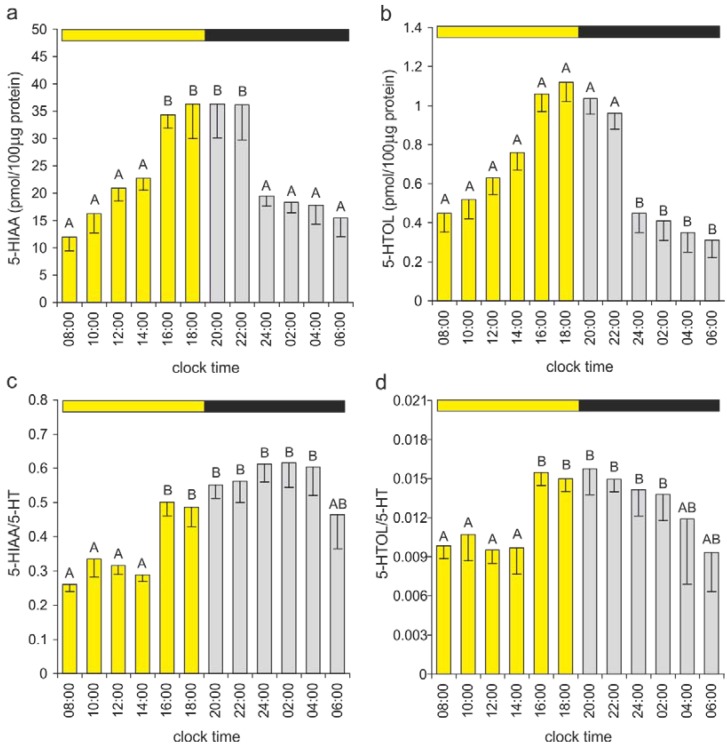
Indoles formed by the oxidative deamination of 5-HT. (**a**,**b**) The 5-HIAA and 5-HTOL contents and (**c**,**d**) the 5-HIAA/5-HT and 5-HTOL/5-HT ratios in the duck pineal organs taken at two-hour intervals over a daily cycle (means and standard errors, *n* = 5). For explanations, see Figure 5.

Our study showed that a large portion of 5-HT in the duck pineal organ underwent oxidative deamination, resulting in 5-HIAA and 5-HTOL formation (Figure 4). In this organ, similar to other biological systems [57], the conversion of 5-hydroxyindole acetaldehyde to 5-HIAA was much more intensive than the conversion to 5-HTOL (Figure 4). The 5-HIAA and 5-HTOL levels changed significantly during the light-dark cycle, and the patterns of these fluctuations were similar to the 5-HT rhythm (Figure 6a,b). However, the ratios of 5-HIAA/5-HT and 5-HTOL/5-HT (Figure 6c,d) were significantly lower between 08:00 and 14:00 h than between 16:00 and 04:00 h (5-HIAA/5-HT) or between 16:00 and 02:00 h (5-HTOL/5-HT). These data, together with information concerning the 5-HT rhythm, suggest that the rate of 5-HT oxidation is diminished during the first half of the photophase compared with other portions of the daily cycle. Most likely, the reduced metabolism of 5-HT helps to restore the pool of this amine after the nighttime decline. Interestingly, no significant changes in the 5-HIAA/5-HT and 5-HTOL/5-HT ratios were observed during the scotophase, despite the large drop in the 5-HT content. Positive correlations between the contents of 5-HT and 5-HIAA have been reported in several species [23,25,44,45,58,59]. The pineal content of 5-HTOL was infrequently measured; however, a close relationship with the 5-HT level was also found [35,60]. In conclusion, oxidative deamination should be considered an extremely important component in regulation of the pineal 5-HT content. Unfortunately, the factors controlling the intensity of this reaction remain unknown. The biological significance of 5-HT oxidative deamination in the pineal is also enigmatic, and the question of why such large amounts of 5-HIAA are produced by the pineal organ, even at night, awaits an answer.

The 5-MTAM level did not exceed 0.15% of the investigated indoles in the duck pineal organ (Figure 4). Therefore, in contrast to the acetylation and oxidative deamination of 5-HT, the process of methylation leading to 5-MTAM formation seems to have no effect on the pineal 5-HT level. The diurnal rhythm of 5-MTAM will be characterized in the next section.

#### 2.2.3. Melatonin and Other 5-Methoxyindoles

In contrast to NAS, the diurnal cycle of the MLT level in the duck pineal (Figure 5e) showed only an *ca.* five-fold difference between the peak (22:00–04:00 h) and nadir (14:00–16:00 h). During the photophase, MLT occurred at an approximately two-fold higher level than NAS (Figure 5f). However, at night, the NAS content exceed the MLT level by more than 50% (Figure 5f). These data suggest that NAS is produced at a level that is beyond the enzymatic capacity of ASMT during the scotophase. Our results provide strong support for the idea that ASMT is a rate-limiting enzyme of night-time MLT synthesis [54,61].

Of note, ASMT activity did not show diurnal fluctuation in the pineal organs of the majority of the investigated species [2,61], including the domestic duck [33]. For many years, it has been believed than the enzymatic capacity of ASMT is adequate to convert a large amount of NAS, which is produced at night, into MLT. Because of this mining, AA-NAT has been considered to be the enzyme that solely controls MLT production. Analyzing the literature data, notably, Zawilska *et al*. [33] reported an *ca.* 3-fold increase in the activity of AA-NAT and an *ca*. 11-fold increase in the MLT content during the scotophase in two-week-old ducks.

**Figure 7:**
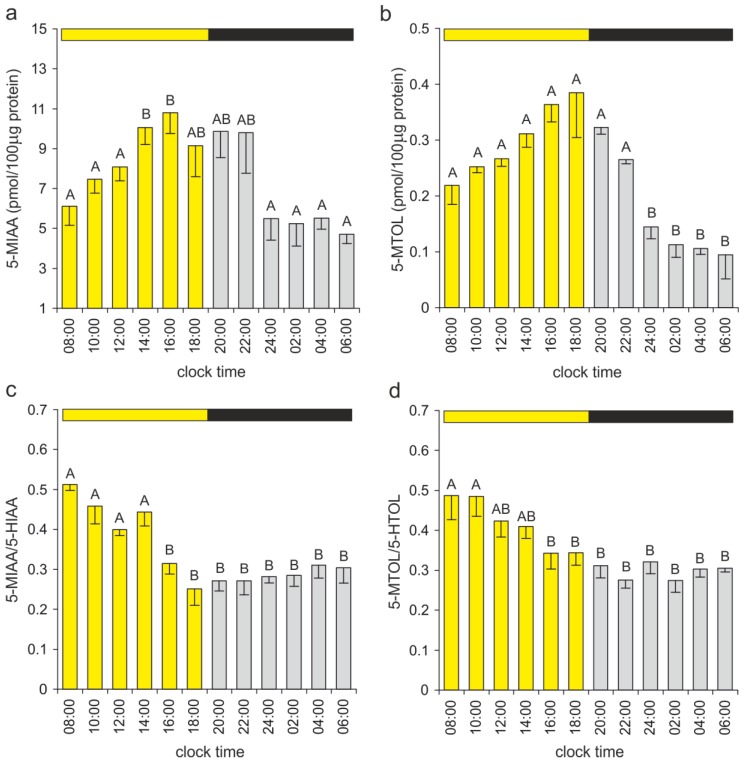
Methylated derivatives of indoles formed by 5-HT oxidative deamination. (**a**,**b**) The 5-MIAA and 5-MTOL contents and (**c**,**d**) the 5-MIAA/5-HIAA and 5-MTOL/5-HTOL ratios in the duck pineal organs taken at two-hour intervals over a daily cycle (means and standard errors, *n* = 5). For explanations, see Figure 5.

The catalytic activity of ASMT also results in the methylation of 5-hydroxyindoles other than NAS and in the formation of 5-MIAA, 5-MTOL, and 5-MTAM. The diurnal rhythms of 5-MIAA and 5-MTOL in the duck pineal organ were almost parallel to the daily changes in their immediate precursors, except for the first half of the photophase, when the 5-MIAA/5-HIAA and 5-MTOL/5-HTOL ratios were higher than during the remaining portion of the daily cycle (Figure 7a−d). The time-dependent differences in the quantitative relationships between products of 5-HT oxidative deamination and their methylated derivatives may be considered a consequence of limitations in the ASMT activity. During the first half of the photophase, the contents of 5-hydroxyindoles were relatively low, and large portions of 5-HIAA and 5-HTOL pools were methylated by ASMT. During the second half of the photophase, these indoles were present in larger amounts, the ASMT activity most likely remained at the same level, and the 5-MIAA/5-HIAA and 5-MTOL/5-HTOL ratios were lower. At night, when the 5-HIAA and 5-HTOL levels dropped again, their methylation was probably limited by competition with high amounts of NAS, and the ratios of 5-MIAA/5-HIAA and 5-MTOL/5-HTOL did not increase. Notably, due to the extremely low level of 5-MTAM, the oxidative deamination of this indole [3] cannot be considered an important source of 5-MIAA and 5-MTOL in the duck pineal organ.

Despite the prominently lower 5-MTOL than 5-MIAA content (approximately 25-fold difference in the duck pineal), 5-MTOL has been much more intensively studied than 5-MIAA. Therefore, the diurnal rhythms of 5-MTOL, with the highest levels during the photophase, were reported in the pineal organs and in the blood plasma of several species [25,32,34,35,44,45,46,62,63], including the domestic duck [33], whereas daily changes in the 5-MIAA level were described in the pineal organs of only a few species (rainbow trout [25], pigeon [35], Japanese quail [64], rat [65], and golden hamster [66]). Studies on Japanese quail demonstrated the presence and diurnal fluctuations of 5-MIAA in blood plasma and identified the pineal organ as a source of this indole in the circulation [64].

**Figure 8:**
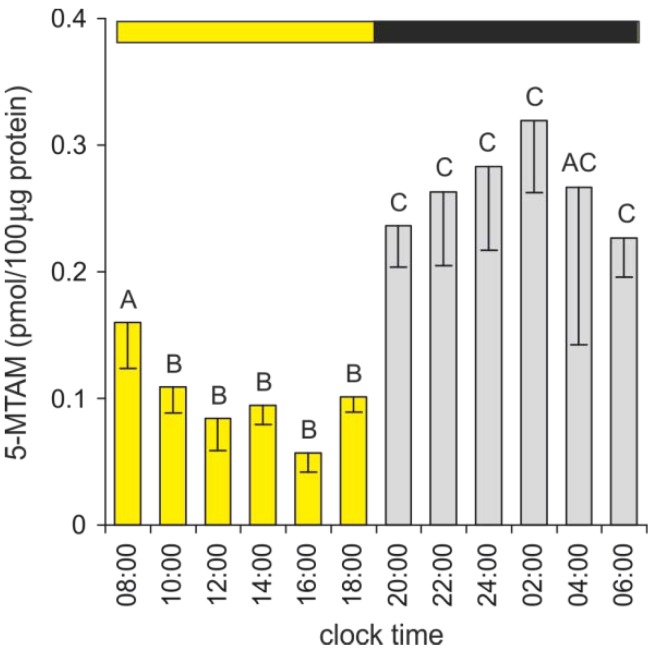
The content of 5-MTAM in the duck pineal organs taken at two-hour intervals over a daily cycle (means and standard errors, *n* = 5). For explanations, see Figure 5.

The pineal level of 5-MTAM was significantly (three-fold) higher during the scotophase than between 10:00 and 18:00 h (Figure 8). In contrast to 5-MIAA and 5-MTOL, the daily rhythm of the 5-MTAM content did not correlate with the changes in the 5-HT level. The synthesis and/or storage of 5-MTAM appears to occur in specific cell compartments (perhaps vesicles) of duck pinealocytes; therefore, the diurnal changes in the 5-MTAM level could be independent of the 5-HT content and transformations of other indoles. Our *in vitro* study (unpublished data) demonstrated that the 5-MTAM level did not increase during the incubation of duck pineal organs in the presence of MLT; however, both 5-MTAM and 5-HT levels dramatically rose after treatment with a monoamine oxidase inhibitor. These results excluded the possibility that 5-MTAM is formed in the duck pineal organ via MLT deacetylation, which is a process known from retina studies [3,67,68].

Due to the extremely low level and limitations of the analytical methods, the pineal content of 5-MTAM has been infrequently studied. Initially, 5-MTAM was detected in rodents only after the administration of MAO inhibitors [69]. Assay improvement enabled the demonstration of the day-night differences in the 5-MTAM content, with higher levels during the photophase, in the rat and quail pineal organs [70]. Recently, diurnal changes in the 5-MTAM content were reported in rainbow trout [25]. Two daily peaks in the pineal 5-MTAM level were observed in the fish exposed to 12L:12D and 8L:16D, either at the beginning or at the end of the night [25]. In contrast, in trout kept under a 16L:8D photoperiod, the 5-MTAM peak occurred at night [25]. The biological activity of 5-MTAM has been well documented [70]. However, low 5-MTAM content and its susceptibility to degradation [69,71] led to the proposal that this biogenic amine plays a role as a paracrine regulator of pineal activity rather than of the pineal hormone. 

### 2.3. Catecholamines and Their Metabolites

The second aim of our study was to determine whether the contents of NE and DA, of their precursor, DOPA, and of their metabolites show diurnal fluctuations in the duck pineal organ. We were interested in the following two questions: (1) Which of these substances can be used as a valid indicator of daily changes in the activity of the pineal sympathetic input in post-mortem studies? (2) Does the adrenergic innervation provide information concerning the light-dark cycle to the duck pineal organ?

**Figure 9:**
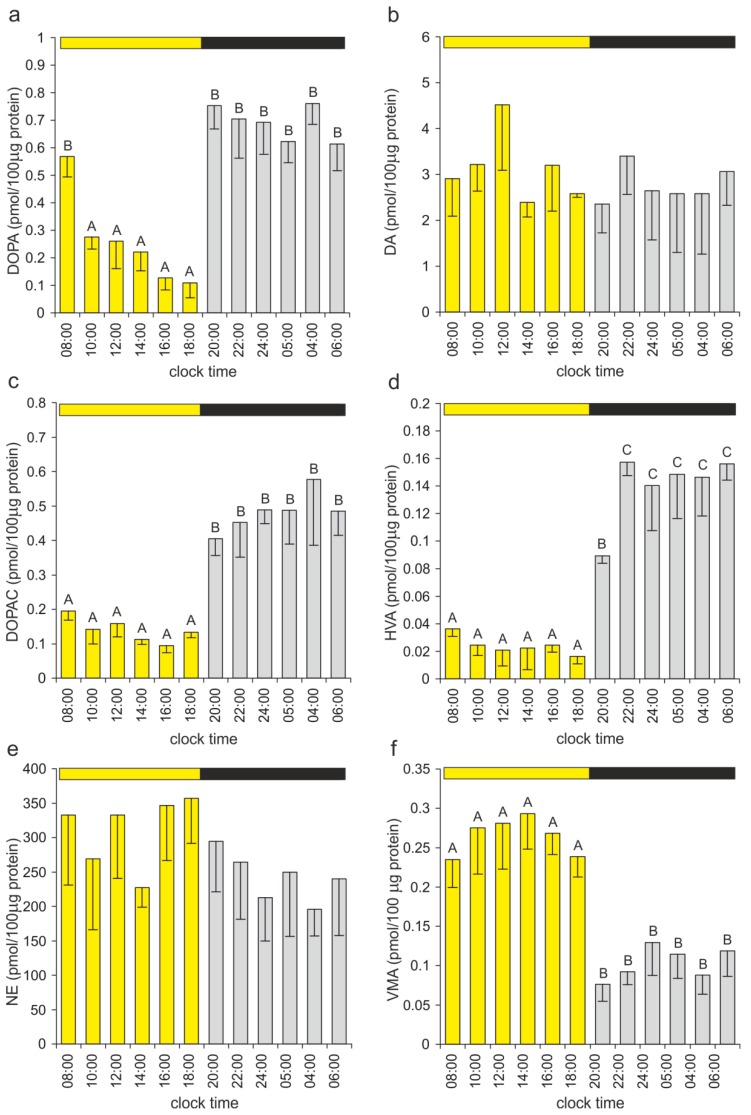
Catecholamines and their metabolites. (**a−f**) The DOPA, DA, DOPAC, HVA, NE and VMA contents in the duck pineal organs taken at two-hour intervals over a daily cycle (means and standard errors, *n* = 5). For explanations, see Figure 5.

The DOPA content in the duck pineal organs was significantly higher during the night than during the day (Figure 9a). The DOPA levels in the organs taken before (at 18:00 h) and after (at 20:00 h) the onset of darkness differed more than seven-fold. No significant differences in the DA content were noted (Figure 9b). The levels of the DA metabolites DOPAC and HVA were significantly higher during the scotophase than during the photophase (Figure 9c,d). The nighttime DOPAC and HVA contents exceeded the daytime values by approximately 350% and 600%, respectively. The NE levels varied between the investigated pineal organs; however, these differences were not statistically significant (Figure 9e). The VMA content was significantly more than two-fold higher during the photophase than during the scotophase (Figure 9f).

According to our results, the NE and DA contents showed no significant diurnal changes in the duck pineal organ. In the studies performed by Cassone *et al*. [36] on 12-week-old chickens, similar results were reported for NE, whereas a small peak of DA was noted shortly after the onset of darkness. In contrast, Zawilska *et al*. [72] found rhythmic changes in the pineal NE content, with levels in the dark period that were markedly higher than in the light period in chickens at 30 and 57 days old, whereas no daily variations were observed in younger birds. The pineal NE content markedly increased in chickens during the first two months of life [72]. This elevation is obviously related to the postembryonic development of the sympathetic innervation of the avian pineal organ [73]. Variable results were obtained in rodent investigations, depending on the species and on the length of the photoperiod. The lack of diurnal fluctuations in the NE level was observed in Syrian hamsters kept under short, equal and long photoperiods [23,24,44] and in Djungarian hamsters housed under a long photoperiod [45]. The NE content was higher at night than during day in the pineal organs of rats maintained under a 12L:12D cycle [24] and in Djungarian hamsters under a 10L:14D cycle [45]. The amplitudes of diurnal changes in both species were low. In contrast to these data, a robust rhythm of NE release in the rat pineal was demonstrated using a microdialysis method [74]. The contents of DA and its acid metabolite, DOPAC, presented rhythmical changes, with higher levels at night, in all studied rodents [23,24,44,45]. Analyzing the above presented data, notably, NE and DA are stored in vesicles in nerve fibers, and only a portion of these vesicles is released during signal transmission. Therefore, the changes in the neurotransmitter contents most likely do not reflect the diurnal fluctuations in their release. 

In contrast to DA and NE, the levels of their metabolites showed prominent diurnal rhythms in the duck pineal organ. The DOPAC and HVA contents were higher during the scotophase than during the photophase, and these data suggest that DA is released from the nerve endings primarily at night. The opposite rhythm was observed for the VMA level and strongly confirms the widespread, but not fully documented, idea that NE is released from the nerve fibers in the avian pineal gland during the daytime. Previously, this concept was supported by studies regarding NE turnover in the chicken pineal organ [36] and by the results of experiments demonstrating the inhibitory action of NE on MLT secretion in avian pinealocytes [17]. In contrast to NE, the role of DA in the pineal organ of birds is unknown, and its action at both presynaptic and postsynaptic levels should be considered. However, notably, the DA content in the duck pineal organ is approximately 100-fold lower than NE. Unfortunately, the chemical characteristics of sympathetic nerve fibers in the pineal organ of birds, in contrast to mammals [75,76,77], are completely unknown, and this deficiency limits the interpretation of biochemical data.

Our results show that DOPAC, HVA and VMA should be considered valid markers of the sympathetic input activity in the avian pineal organ. The only limitation regarding the use of these compounds as neuronal transmission indicators is their low pineal content. The shifts from daytime to nighttime levels of the NE and DA metabolites were rapid and occurred exactly at the time of the light-dark transition. These patterns of changes suggest the sympathetic innervation as a source of precise information concerning the light-dark cycle in the duck pineal organ.

We also noted a prominent rhythm of the DOPA content in this organ. However, the current stage of knowledge concerning the pineal innervation does not provide an explanation of this phenomenon. It is reasonable to suspect that diurnal fluctuations in DOPA, DOPAC and HVA contents have their source in daily changes in the activities of dopaminergic, non-adrenergic intra-pineal nerve fibers.

## 3. Experimental Section

### 3.1. Animal Experimental Procedures

This study was performed on 60 females of the domestic duck (*Anas platyrhynchos f. domestica*) hatched in April and housed under a cycle of a 12 h photophase and 12 h scotophase, starting from the first day of postembryonic life. During the photophase, from 07:00 to 19:00 h, full-spectrum fluorescent lamps provided illumination, with a light intensity of 300 lux at the floor level. The animals were kept in complete darkness during the scotophase, except for the first two weeks, when infrared brooding lamps were permanently turned on. The birds had free access to standard food and water. At 14 weeks old, the ducks were killed at two hour intervals. The pineal organs were quickly removed and frozen at −75 °C. During the scotophase, all procedures were performed in total darkness using noctovision goggles. The protocol for animal experiments was reviewed and approved by the Local Ethical Commission in Olsztyn, Poland.

### 3.2. Analytical Procedures

#### 3.2.1. Chemicals

The following chemicals of the highest purity were purchased from J. T. Baker Chemicals (Center Valley, PA, USA) and used for preparing mobile phases: sodium acetate, sodium dihydrogen phosphate, disodium EDTA, 1-octanesulfonic acid sodium salt, citric acid, acetic acid, and phosphoric acid. Methanol and acetonitrile, which were both of gradient-grade HPLC purity, were provided by Merck Millipore (Billerica, MA, USA). TRP, 5-HTRP, 5-HT, NAS, MLT, 5-HIAA, 5-MIAA, 5-MTOL, 5-MTAM, NE, DOPA, DA, DOPAC, HVA, VMA, epinephrine, and perchloric acid were obtained from Sigma-Aldrich (St. Louis, MO, USA). 5-HTOL was provided by Santa Cruz Biotechnology (Dallas, TX, USA). Sodium hydroxide was purchased from POCH (Gliwice, Poland), and a Bradford protein assay kit was purchased from Bio-Rad (Hercules, CA, USA). Ultrapure water (18.2 MΩ, TOC ≤ 5 ppb), which was freshly prepared using a Milli-Q integral purification system (Merck Millipore, Billerica, MA, USA), was used in all procedures.

#### 3.2.2. Sample Preparation for Indole and Catecholamine Assays

The frozen pineal organs were weighed and sonicated (5 × 2 s, 1 W) in 100 µL of ice cold 0.1 M perchloric acid using a Vibra-Cell VC 70 ultrasonic processor equipped with a 2 mm probe (Sonics & Materials Inc., Newtown, CT, USA). The homogenate was incubated for 20 min in an ice-bath and then centrifuged at 60,000 g and 4 °C for 15 min (Allegra 64R, Coulter Beckman, Indianapolis, IN, USA). The supernatant was carefully transferred into autosampler vial and the pellet was frozen at −75 °C for protein assay. The indole and catecholamine assays were completed within four hours after sample preparation.

#### 3.2.3. Indole Assay

The indole assay was performed using an Ultimate 3000 chromatographic system (Dionex, Sunnyvale, CA, USA), which consisted of a LPG 3400A pump with a built-in four-channel degasser, WPS 3000SL autosampler, TCC 3100 column thermostat and FLD 3400 RS fluorescence detector. The system was controlled using Chromeleon 6.8 software (Dionex, Sunnyvale, CA, USA). The injection volume of the standards and samples was 20 µL. The separation was performed at a temperature of 30 °C on a Hypersil Gold aQ column with 3-µm particle size and with dimensions of 150 × 4.6 mm, protected by a dedicated guard column (Thermo Scientific, Waltham, MA, USA). The mobile phase was composed by on-line mixing of methanol and a water solution of 5 mM sodium acetate and 0.01 mM disodium EDTA (pH = 4.5 by addition of acetic acid). The initial concentration of methanol was 10% (*v*/*v*). Next, between the 9th and 20th min of the separation, the methanol content was linearly increased to 30% (*v*/*v*) and then it was kept at a constant level for 18 min. The methanol content was decreased to the initial value between the 38th and 40th min of the separation, and after 5 min equilibration, the system was ready for the next injection. The flow rate of the mobile phase was 1 mL/min. The detection was performed at an excitation wavelength of 280 nm and at an emission wavelength of 345 nm, with a constant temperature of 45 °C. The sensitivity of the detector was changed at the beginning of the 4th min of the separation from level 6 to level 2; at the beginning of the 5th min, to level 4; and at the beginning of the 7th min, to level 8. The chromatograms were analyzed using Chromeleon 6.8 software (Dionex, Sunnyvale, CA, USA).

#### 3.2.4. Validation of the Indole Assay

To determine the effect of perchloric acid on the indole content, the glands were sonicated in water, and one portion of the homogenate was deproteinized by adding the acid and another portion was deproteinized by filtration through a molecular filter with a 3-kDa molecular weight cut-off (Sigma-Aldrich, St. Louis, MO, USA). The stability of indoles in the pineal homogenate prepared in 0.1% perchloric acid was determined by assaying the samples immediately after centrifugation and after 1, 2, 4, 6, 12 and 24 h of the storage at 21 °C. At the chromatographic step, the validation procedures involved: (1) modification of the gradient slope during the separation of standards and pineal homogenates; (2) assay of standards in ten replicates on the same day or during ten consecutive days; (3) assay of pineal homogenates before and after adding exogenous indoles; and (4) comparison of the results of 5-hydroxyindole measurements obtained with this method and with the method using electrochemical detection, as described below.

#### 3.2.5. Assay of Catecholamines and Their Metabolites

The contents of catecholamines and their metabolites were measured using a chromatographic system composed of a LPG 3400M four-channel pump with a built-in degasser (Dionex, Sunnyvale, CA, USA), WPS 3000SL autosampler (Dionex, Sunnyvale, CA, USA) and CoulArray 5600A electrochemical detector equipped with two four-channel 6210 coulometric cells (ESA Inc., Chelmsford, MA, USA). The system was controlled by Chromeleon 6.8 (Dionex, Sunnyvale, CA, USA) and CoulArray 3.10 Data Station (ESA Inc., Chelmsford, MA, USA) software. Standards or samples were injected at 20-μL volumes onto the MG 150 × 3.2 mm i.d. column, which was filled with 3-µm C18 particles (ESA, Inc., Chelmsford, MA, USA). The column and coulometric cells were kept at 25 °C. The mobile phase consisted of acetonitrile and a buffer containing 90 mM sodium phosphate dihydrate, 50 mM citric acid, 1.7 mM 1-octanesulfonic acid sodium salt and 50 µM disodium EDTA (pH 3.05 with phosphoric acid), mixed together in a proportion of 6:94 (*v*/*v*). The mobile phase was pumped at a flow rate of 0.5 mL/min. The potentials applied on consecutive electrodes were as follows: −150, 200, 350 and 450 mV. The data acquisition and integration of chromatograms were performed using CoulArray 3.10 Data Station (ESA Inc., Chelmsford, MA, USA) software. The current amperage on a 200 mV electrode was used for determining DOPA, DOPAC, DA and NE contents, and the current amperage on a 350 mV electrode was used for measuring HVA and VMA. The duration of the analysis was 45 min due to the retention time of 5-HT (this component could interfere during the next assay).

#### 3.2.6. Protein Assay

After pineal homogenate centrifugation, the pellet was dissolved in 750 µL of 1 M sodium hydroxide. The obtained solution was diluted 1:1 in water and used for determining the protein content using the Bradford microplate assay. Solutions of bovine serum albumin in 0.5 M sodium hydroxide served as standards for preparing the calibration curve.

### 3.3. Statistical Analysis

The data were analyzed by a one-way ANOVA and by Duncan’s test as a *post-hoc* procedure using Statistica 10.0 software (StatSoft, Tulsa, OK, USA).

## 4. Conclusions

The obtained results show that the contents of all investigated tryptophan derivatives in the duck pineal organ vary during the course of the light-dark cycle. The analysis of the quantitative relationships between indoles points to the hydroxylation of TRP, acetylation and oxidative deamination of 5-HT, and methylation of 5-hydroxyindoles as the extremely important steps determining pineal indole metabolism. The measurements of NE and DA metabolites demonstrated that the sympathetic innervation provides the duck pineal organ with precise information concerning the course of the environmental light-dark cycle. The HPLC indole assay, developed in our study, could be useful in further investigations on MLT biosynthesis, both in the pineal and in the peripheral organs [78,79,80].
